# French multicentre prospective evaluation of radiofrequency ablation in the management of haemorrhoidal disease

**DOI:** 10.1007/s10151-023-02787-1

**Published:** 2023-04-02

**Authors:** A. Laurain, D. Bouchard, J.-M. Rouillon, P. Petit, A. Liddo, B. Vinson Bonnet, A. Venara, J.-M. Didelot, G. Bonnaud, A. Senéjoux, T. Higuero, P. Delasalle, A.-L. Tarrerias, F. Devulder, A. Castinel, C. Thomas, H. Pillant Le Moult, C. Favreau-Weltzer, L. Abramowitz

**Affiliations:** 1 Clinique Blomet Ramsay santé, 136 rue Blomet, 75015 Paris, France; 2 Service de Proctologie, Hôpital Bichat, APHP, 46 Rue Henri Huchard, 75018 Paris, France; 3 Service de Proctologie, Hôpital Bagatelle, 33400 Talence, France; 4 Service de Gastroentérologie, Polyclinique Montréal, Route de Bram, 11000 Carcassonne, France; 5 Clinique Santé Atlantique, Elsan, 44800 Saint Herblain, France; 6 Cabinet médical, 1 Quai du Havre, 59200 Tourcoing, France; 7 Clinique de la Victoire, 1 Quai du Havre, 59200 Tourcoing, France; 8 Service de Chirurgie Digestive CHI Poissy, St Germain, France; 9 Service de Chirurgie Viscérale et Endocrinienne CHU Angers, 4 rue Larrey, Angers, France; 10 Cabinet médical, 1019 Avenue du Pr Louis Ravaz, 34080 Montpellier, France; 11 Clinique Clémentville, 25 Rue de Clémentville, 34070 Montpellier, France; 12 Clinique Ambroise Paré, 31000 Toulouse, France; 13 Centre Hospitalier Privé, 6 Bd de la Boutière, 35760 Saint Grégoire, France; 14 Cabinet médical, 11, bd du général Leclerc, 06240 Beausoleil, France; 15 Clinique Kantys centre, 7 avenue Durante, 06004 Nice, France; 16 Clinique du Palais, 25 Avenue Chiris, 06130 Grasse, France; 17 Clinique du Trocadéro, 75016 Paris, France; 18 SELARL Hépato-gastroentérologie, 89 rue Louis Victor de Broglie, 51430 Bezannes, France; 19 Service de Proctologie, Clinique Tivoli Ducos, 91 rue de Rivière, Bordeaux, France; 20 Service d’Hépato-gastroentérologie, Institut mutualiste Montsouris, 40 boulevard Jourdan, 75014 Paris, France; 21 Service de Proctologie, Hôpital St Joseph rue Losserand, 75014 Paris, France

**Keywords:** Haemorrhoids, Radiofrequency ablation, Surgery, Rafaelo^©^ procedure

## Abstract

**Purpose:**

The aim of this study was to evaluate the efficacy and safety of radiofrequency ablation (RFA) in the management of haemorrhoidal disease with 1 year’s follow-up.

**Method:**

This prospective multicentre study assessed RFA (Rafaelo^©^) in outpatients with grade II–III haemorrhoids. RFA was performed in the operating room under locoregional or general anaesthesia. Primary endpoint was the evolution of a quality-of-life score adapted to the haemorrhoid pathology (HEMO-FISS-QoL) 3 months after surgery. Secondary endpoints were evolution of symptoms (prolapsus, bleeding, pain, itching, anal discomfort), complications, postoperative pain and medical leave.

**Results:**

A total of 129 patients (69% men, median age 49 years) were operated on in 16 French centres. Median HEMO-FISS-QoL score dropped significantly from 17.4/100 to 0/100 (*p* < 0.0001) at 3 months. At 3 months, the rate of patients reporting bleeding (21% vs. 84%, *p* < 0.001), prolapse (34% vs. 91.3%, *p* < 0.001) and anal discomfort (0/10 vs. 5/10, *p* < 0.0001) decreased significantly. Median medical leave was 4 days [1–14]. Postoperative pain was 4/10, 1/10, 0/10 and 0/10 at weeks 1, 2, 3 and 4. Seven patients (5.4%) were reoperated on by haemorrhoidectomy for relapse, and three for complications. Reported complications were haemorrhage (3), dysuria (3), abscess (2), anal fissure (1), external haemorrhoidal thrombosis (10), pain requiring morphine (11). Degree of satisfaction was high (+ 5 at 3 months on a − 5/+ 5 scale).

**Conclusion:**

RFA is associated with an improvement in quality of life and symptoms with a good safety profile. As expected for minimally invasive surgery, postoperative pain is minor with short medical leave.

**Clinical trial registration and date:**

Clinical trial NCT04229784 (18/01/2020).

**Supplementary Information:**

The online version contains supplementary material available at 10.1007/s10151-023-02787-1.

## Introduction

Haemorrhoidal disease is common. Its prevalence in the general population is estimated at 40% [1, 2]. After failure of other treatments, surgery improves the quality of life of patients [3, 4]. The standard surgical technique in Europe, described by Milligan and Morgan in 1937 [5], is the submucosal resection of the three main haemorrhoidal bundles. In the long-term, such haemorrhoidectomy is the most effective [6]. However, the long and painful postoperative course could be a barrier for patients. In addition, minimally invasive surgeries for haemorrhoids have been developed, such as stapled haemorrhoidopexy [7] and Doppler arterial ligation with or without mucopexy [8]. The concept of minimally invasive surgery is to treat internal haemorrhoids by intervening only above the dentate line, which limits postoperative pain. The use of a radiofrequency current in the treatment of haemorrhoidal disease is an innovative technique. It consists in delivering 4 MHz radiofrequency energy into the haemorrhoidal vascular tissue by means of rigid electrodes. The energy is converted into heat to destroy haemorrhoidal vessels and initiate a process of submucosal fibrosis. Although radiofrequency ablation (RFA) by the Rafaelo^©^ procedure is already used in many European countries, there are only few studies on this minimally invasive technique [9–14]. Most of them are retrospective with low numbers of patients and conducted in one or two centres. Therefore, the GREP (Proctology Research Group for the French Society of Proctology, SNFCP) and the CREGG (Hepato-Gastroenterology Cabinets and Groups Reflection Club) decided to evaluate RFA in a prospective French multicentre study with 1 year’s follow-up.

## Methods

### Study design

This is a multicentre, national, prospective, open and longitudinal study.

### Setting

Patients were included in 16 French proctology clinics between January 2019 and March 2020. Recruitment was halted as a result of the pandemic. Patients had a preoperative visit and postoperative visits at 1, 3, 6 and 12 months after surgery.

### Participants

The inclusion criteria were age between 18 and 75 years old, and grade II or III haemorrhoidal disease after failure of medico and non-surgical treatment. For patients on anticoagulants or antiplatelet agents (other than aspirin), a short break was recommended in accordance with recommendations [15]. Exclusion criteria were patients with chronic inflammatory disease, anal fissure, anal fistula, external haemorrhoid disease, haematological disease with hemorrhagic risk, patients unable to interrupt anticoagulants or antiplatelet agents, pregnant or breastfeeding women and patients with pacemakers.

### Variables

Like Watson et al. [16] in their randomised study, we decided to choose as primary outcome a quality-of-life score rather than a score focusing solely on symptoms. In effect, surgery is mainly chosen to improve quality of life rather than anatomical correction. Therefore, we chose as our main endpoint the improvement of the HEMO-FISS-QoL score 3 months after RFA. This validated score for evaluation of the burden of haemorrhoidal disease [17] is a self-administered questionnaire of 23 questions covering four dimensions: physical disorders, psychology, defecation and sexuality. We analysed the total HEMO-FISS-QoL score as well as each dimension of the score. Scale results range from 0 (low burden) to 100 (very high burden). Secondary criteria were evolution of the HEMO-FISS-QoL score at 1, 6 and 12 months as well as haemorrhoidal symptoms at 1, 3, 6 and 12 months after surgery. Haemorrhoidal symptoms were rated as “present” or “absent” for prolapse, bleeding, pain and pruritus ani. If a symptom was present, it was evaluated by the Goligher scale for prolapse, by the degree of severity for bleeding (minimal, moderate, severe), by a visual analogue scale (VAS) from 0 to 10 for pain and on a scale of 0 to 10 for pruritus ani. Global anal discomfort was rated from 0 to 10. We also evaluated time of hospitalisation and medical leave (or the number of days of limitation of activities for unemployed persons). To limit bias, medical leave certificates were given for 3 days after surgery and extended for periods of 3 days at the patient’s request. We evaluated safety by reporting complication and anal incontinence. Using a self-reported questionnaire, we evaluated maximal and mean postoperative pain on a 0–10 scale every day during the first month and use of analgesics. Patient satisfaction was assessed on an − 5/+ 5 scale for the evolution of haemorrhoidal disease and the degree of satisfaction 1 year after surgery.

### Surgery

**Fig. 1:**
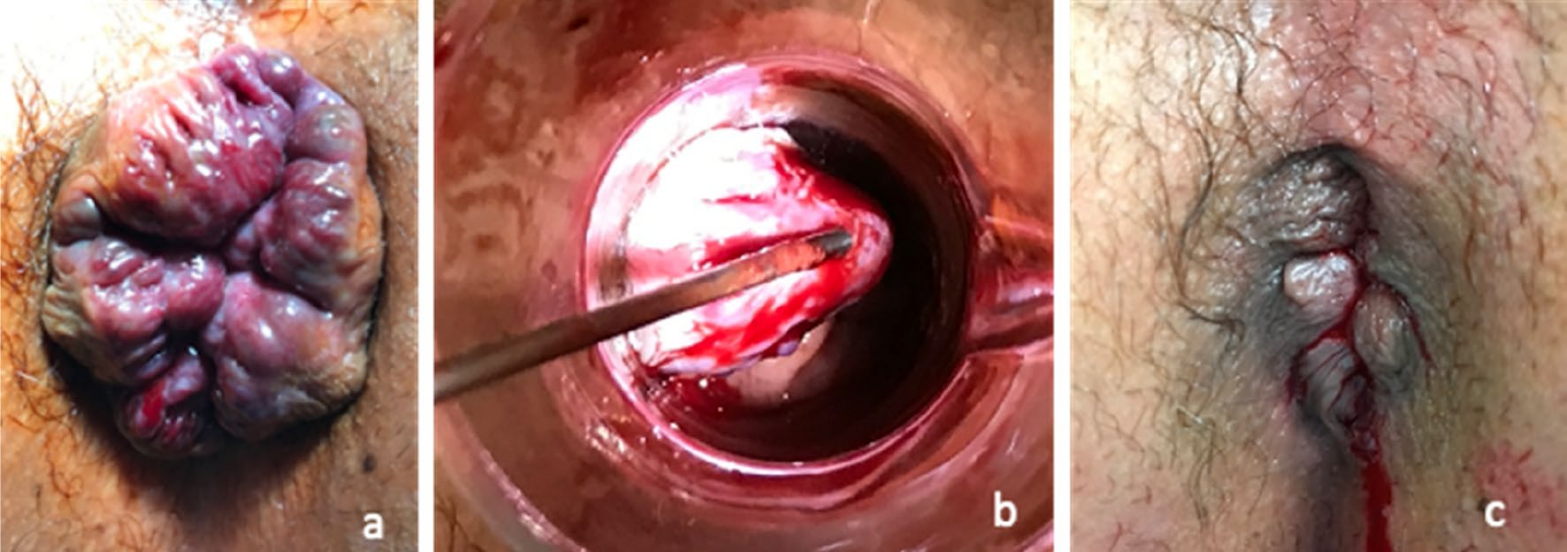
Surgical procedure. **a** Preoperative view. **b** Insertion of the HPR45i probe into the haemorrhoidal bundle 1 cm above the dentate line and tilting of the haemorrhoid away from the sphincter. **c** Postoperative view

Surgeons had to have performed at least five procedures before inclusion. The procedure consists in delivering a current of 4 MHz radiofrequency waves by a Rafaelo^©^ generator (CE marking, F Care Systems) at low temperature by microfibre electrodes using a single-use disposable needle (HPR45I probe, CE1304). The procedure is performed under general or locoregional anaesthesia. A perineal block is performed (20 ml of ropivacaine [4]), then 1% xylocaine is injected under the internal haemorrhoidal bundle to lift it from the internal sphincter. The probe is inserted into the haemorrhoidal bundle 1 cm above the dentate line and tilted away from the internal sphincter (Fig. 1). Pulses of 10–20 s (for a maximum of 2000 J per bundle without exceeding a total of 6000 J) are delivered while progressively removing the needle and are stopped at early appearance of whitening around the needle. The haemorrhoidal bundle is then cooled with a compress soaked in ice-cold saline. This process is repeated depending on the patient’s anatomical condition. Total joules and time of procedure were recorded.

### Postoperative prescription

Postoperative prescriptions were standardised according to French recommendations and included [4] metronidazole (1.5 g/day) and ketoprofen (200 mg/day) for 7 days; macrogol, suppositories (carrageenan and titanium dioxide) and cream (dexpanthenol) until healing. In case of pain, paracetamol (500 mg), codeine (30 mg) or tramadol (LP 150 mg), if necessary, were prescribed.

### Study size

When writing the protocol (2018), we did not have enough data to be able to calculate a study size. As this study was considered as proof of concept, we arbitrarily chose an objective of 150 patients, which seemed achievable in about 1 year of recruitment and enough to evaluate a few associated factors.

### Statistical considerations

For qualitative data, this included the number of completed and missing data as well as the frequency and percentage (referring to filled data) for each modality. Proportions were estimated with their exact 95% CIs when appropriate. Data were compared using the chi-squared test or Fisher’s exact test, according to expected values under the assumption of independence. For quantitative data, this included number of filled and missing data, arithmetic mean, standard deviation, median, first and third quartiles, minimum and maximum. Data were compared using the Student or Mann–Whitney–Wilcoxon tests depending on variable distribution.

A Firth’s penalised likelihood multivariate logistic regression was used to measure the probability for a Milligan–Morgan treatment, adjusted for gender, age, indication for surgery, grade of haemorrhoids, HEMO-FISS-QoL score at month 0, anal discomfort at month 0, prolapse and bleeding.

A Firth’s Penalised Likelihood multivariate logistic regression was used to measure the probability of having a mean daily pain of ≥ 6 at least once after surgery and/or level-3 analgesic, adjusted for gender, age, indication for surgery, grade of haemorrhoids, HEMO-FISS-QoL score at month 0, anal discomfort at month 0, prolapse and bleeding.

All comparisons were performed at a statistical significance level set at *p* < 0.05.

All calculations were made using SAS for Windows (v 9.4; SAS Institute Inc).

### Ethics approval statement

The study protocol was validated by the Ile de France Ethics Committee. The MR 001 reference methodology was applied for CNIL (Commission Nationale de l’Informatique et des Libertés) assessment. The protocol design, analysis of results and drafting of the article were carried out independently from sponsors. Written informed consent forms were collected from patients.

The trial was registered as NCT04229784 (18 January 2020).

## Results

### Characteristics of population and surgery

Table 1 summarises the characteristics of the patients and surgery.

**Table 1:** Patient and surgery characteristics (*N* = 129)

Median age, years (min–max)	48.8 (19–75)
Sex	
Female, *n* (%)	40 (31%)
Male, *n* (%)	89 (69%)
Haemorrhoidal severity (Goligher classification)	
Grade I, *n* (%)	2 (1.6%)
Grade II, *n* (%)	53 (41.1%)
Grade III, *n* (%)	70 (54.3%)
Grade IV, *n *(%)	4 (3.1%)
Patients with antiaggregant or anticoagulation treatment	
Aspirin, *n* (%)	3 (2.3%)
Clopidogrel, *n* (%)	3 (2.3%)
Direct-acting oral anticoagulants, *n* (%)	4 (3.1%)
Vitamin K antagonist, *n* (%)	0
Reasons for surgery (multiple answers)	
Bleeding	106 (82.2%)
Prolapse	79 (61.2%)
Pain	29 (22.5%)
Pruritus ani	16 (12.4%)
Median preoperative HEMO-FISS-QoL* score (min–max)	17.4/100 (0–86)
Patients with dyschezia	17 (13.5%)
Median time of surgery (IQR), minutes	15 (10–25)
Median numbers of joules delivered (IQR)	3925 (2975–4975)
Outpatient surgeries, *n* (%)	122 (97%)
Median time of medical leave, days (IQR)	4 (1–14)

*IQR *interquartile range

*HEMO-FISS-QoL score is a validated quality of life score for evaluation of the burden of haemorrhoidal or fissure disease. It is a self-assessment questionnaire of 23 questions. The scale ranges from 0 (low burden) to 100 (very high burden)

**Fig. 2:**
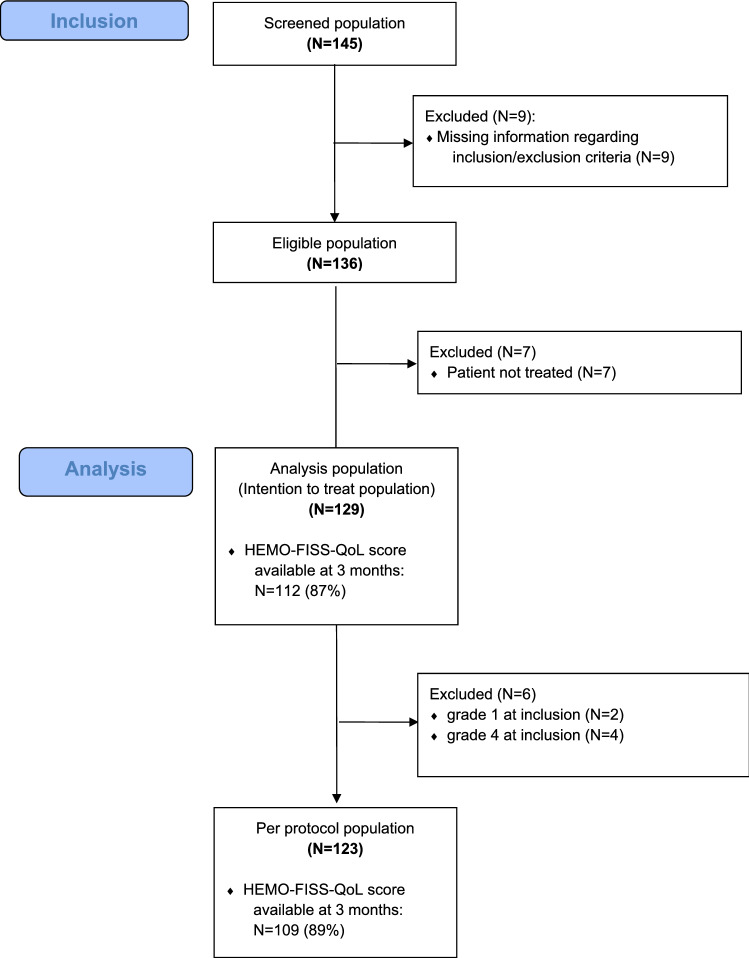
Study flowchart

Between January 2019 and March 2020, 129 patients were prospectively included in 16 private or public French centres by 19 proctologists with 1 year’s follow up (Fig. 2). HEMO-FISS-QoL score was available at 3 months for 112 patients (87%). Median patient age was 49 years old; 89 patients (69%) were male. Haemorrhoidal severity according to the Goligher classification was grade III for 70 patients (54.3%) and grade II for 53 patients (41.1%). A few patients with grade I (two patients) or IV (four patients) were included erroneously. The median number of haemorrhoidal bundles per patient was 3 (IQR 3–3). Clopidogrel and direct-acting oral anticoagulants were taken by three and four patients respectively. The main reasons for surgery were bleeding (106 patients, 82.2%) and prolapse (79 patients, 61.2%).

Surgeries were outpatient (122 patients, 97%) or overnight (4 patients, 3%). Median time of surgery was 15 min (IQR 10–25) with a median number of joules delivered of 3925 (IQR 2975–4975). The median number of bundles treated per patient was 3 (IQR 3–4).

### Efficiency of radiofrequency surgery

#### Evolution of HEMO-FISS-QoL score and haemorrhoidal symptoms

**Fig. 3:**
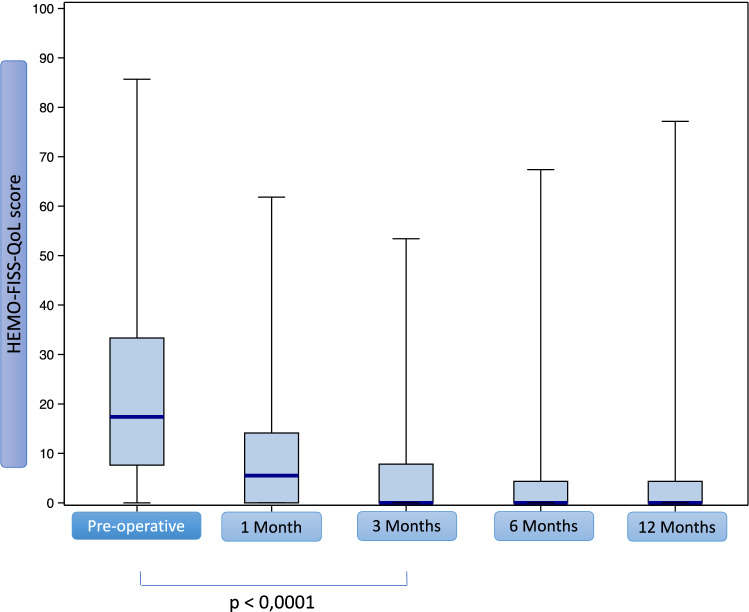
Box plot of HEMO-FISS-QoL score before surgery and 1, 3, 6 and 12 months after surgery

With regard to the main assessment criterion in the intention to treat population (*N* = 129), median HEMO-FISS-QoL scores decreased significantly 3 months after surgery (17.4/100 to 0/100, *p* < 0.0001) (Table 2 and Fig. 3). The median HEMO-FISS-QoL score remained at 0/100 at 6 and 12 months after surgery. The rate of patients with prolapse decreased significantly at 3 and 12 months (91% vs. 34% and 22%, *p* < 0.001). At 12 months, only six patients reported grade III haemorrhoids. The rate of patients reporting bleeding significantly decreased to 84% vs. 22% at 3 months and 12 months (*p* < 0.001). At 12 months, no patient reported severe bleeding and only six reported moderate bleeding. Median global anal discomfort decreased from 5/10 to 0/10 at 3 and 12 months after surgery (*p* < 0.0001).

**Table 2:** Evolution of the HEMO-FISS-QoL score and symptoms of haemorrhoidal disease before surgery and 1, 3, 6 and 12 months after surgery

	Preoperative	1 month	3 months	6 months	12 months
Median HEMO-FISS-QoL score (IQR)	17.4/100 (7.6–33.3)	5.5/100 (0–14.1)	0/100 (0–7.8)	0/100 (0–4.3)	0/100 (0–4.3)
Number of patients with available score (%)	*N* = 127 (98%)	*N* = 120 (93%)	*N* = 113 (88%) *p* < 0.0001	*N* = 99 (77%)	*N* = 114 (88%) *p* < 0.0001
Median HEMO-FISS-QoL score by dimension					
Defecation (IQR)	41.7/100 (16.7–58.3)	16.7/100 (0–25.0)	0/100 (0–16.7)	0/100 (0–16.7)	
Physical troubles (IQR)	18.2/100 (5.6–32.5)	4.5/100 (0–15.9)	0/100 (0–5)	0/100 (0–2.5)	0/100 (0–2.3)
Psychology (IQR)	7.1/100 (0–25)	0/100 (0–8.3)	0/100 (0–0)	0/100 (0–0)	0/100 (0–0)
Sexuality (IQR)	12.5/100 (0–25)	0/100 (0–12.5)	0/100 (0–0)	0/100 (0–0)	0 /100 (0–0)
Existence of prolapse (yes vs. no)	116/127 (91%)	40/114 (35.1%)	35/104 (34%) *p* < 0.001	22/91 (24%)	25/112 (22%) *p* < 0.001
No prolapse	11 (8.6%)	74 (64.9%)	69 (66.3%)	69 (75.8%)	87 (77.7%)
Grade I	1 (0.8%)	12 (10.5%)	12 (11.5%)	7 (7.7%)	6 (5.4%)
Grade II	43 (33.9%)	22 (19.3%)	14 (13.5%)	10 (11%)	13 (11.6%)
Grade III	67 (52.8%)	4 (3.5%)	6 (5.8%)	4 (4.4%)	6 (5.3%)
Grade IV	5 (3.9%)	2 (1.75%)	3 (2.9%)	1 (1.1%)	0
Existence of bleeding (yes vs. no)	107/127 (84%)	24/113 (21.2%)	24/107 (22%) *p* < 0.001	14/95 (15%)	25/113 (22%) *p* < 0.001
No bleeding	20 (16%)	89 (79.5%)	83 (79.0%)	81 (85.3%)	88 (78.6%)
Minor bleeding	31 (24.8%)	17 (15.2%)	17 (16.2%)	11(11.6%)	18 (16.1%)
Moderate bleeding	44 (35.2%)	4 (3.6%)	5 (4.8%)	3 (3.2%)	6 (5.4%)
Severe bleeding	30 (24%)	2 (1.8%)	0 (0%)	0 (0%)	0 (0%)
Median (IQR) of global anal discomfort (scale from 0 to 10, 0 = no discomfort and 10 = maximal discomfort)	5/10 (4–7)	1/10 (0–2)	0/10 (0–2) *p* < 0.0001	0/10 (0–1)	0/10 (0–2) *p* < 0.0001
Pruritus ani (yes vs. no) Median (IQR) (scale from 0 to 10, 0 = no pruritus and 10 = maximal pruritus)	28/126 (22.2%) 4/10 (3–5)	12/111 (10.8%) 3/10 (2.5–5)	14/107(13.1%) 3/10 (2–4)	13/95 (13.7%) 3/10 (2–3)	15/113 (13.3%) 3/10 (2–4)
Median of anal incontinence (IQR) (scale from 0 to 10, 0 = no incontinence, 10 = maximal incontinence)	0/10 (0–0)	0/10 (0–0)	0/10 (0–0)	0/10 (0–0)	0/10 (0–0)
For patients with an incontinence anal score > 0					
* N*	*N* = 9	*N* = 8	*N* = 2	*N* = 1	*N* = 3
Median Wexner score (IQR)	6/20 (3–9)	4.5/20 (2–6)	9/20 (5–13)	4/20 (4–4)	3/20 (3–6)
Terminal dyschezia (yes vs. no)	17/126 (13.5%)	7/110 (6.4%)	7/107 (6.5%)	11/95 (11.6%)	12/113 (10.6%)
Pain (yes vs. no)	42/127 (33.1%)	33/114 (28.9%)	11/110 (10.0%)	9/96 (9.4%)	10/113 (8.8%)
If yes, median pain (IQR) (scale from 0 to 10, 0 = no pain, 10 = maximal pain)					
Median (IQR) of the pain at the time of questioning	2/10 (0–3)	2/10 (1–3)	3/10 (2–4)	1/10 (0–2)	2/10 (1–4)
Median (IQR) of the pain in the last 48 h	3/10 (2–4)	2/10 (2–4)	3/10 (2–5)	2/10 (1–2)	2/10 (1–3)
Median (IQR) of the maximal pain in the last 48 h	5/10 (3–6)	3/10 (3–6)	DM	DM	2/10 (1–3)
Evolution of haemorrhoidal pathology assessed by patients on a − 5/+ 5 scale. Median (IQR)	NA	+ 4 (2–5)	+ 4 (2–5)	+ 4 (3–5)	+ 4 (3–5)
Patient satisfaction assessed by patient on a − 5/+ 5 scale. Median (IQR)	NA	+ 5 (3–5)	+ 5 (3–5)	+ 5 (4–5)	+ 4 (3–5)

*IQR* interquartile range, *NA* not applicable, *MD* missing data

In the per protocol population (without grade I and IV patients, *N* = 123) results are similar to those in the intention to treat population. Median HEMO-FISS-QoL scores significantly decreased 3 months after surgery (from 16.5/100 to 0/100, *p* < 0.0001).

As one centre accounted for 31% of patients, we checked that there was no centre effect (see supplementary table).

### Patient satisfaction

The evolution of haemorrhoidal disease and the degree of satisfaction were, on average, at + 4 and + 5 at 3 and 12 months after surgery on a − 5/+ 5 scale respectively (Table 2). To the question “If it had to be done again, would you agree to be operated on using the same procedure?” 99 patients (86%) answered “yes”. To the question “Would you recommend this operation to relatives?” 94 patients (80%) answered “yes” and 14 patients (12%) answered “perhaps”.

### Medical leave time

Medical leave time was a median of 4 days (IQR 1–14; min–max 0–45) and 8 days on average.

### Instrumental or surgical re-treatment of haemorrhoids during follow-up

A new infrared treatment of haemorrhoids was carried out in three patients (2.3%). Seven patients (5.7%) were reoperated on during follow-up, all by haemorrhoidectomy using the Milligan–Morgan technique (technique was left to the investigator).

### Postoperative pain and analgesics consumption

The median maximum postoperative pain reported was 4/10 (IQR 1–7) during the first week, it decreased to 1/10 (IQR 0–4) the second week, to 0/10 (IQR 0–2) the third week and to 0/10 (IQR 0–1) the fourth week. The median number of level-2 analgesic tablets consumed per day was 0 at weeks 1, 2, 3 and 4. During the first week, 50 patients (42.4%) took at least one level-2 analgesic. Eleven patients (8.5%) had intense pain requiring level-3 analgesics (morphine).

### Safety

No perioperative complications were reported. Postoperative complications reported included three postoperative haemorrhages 5, 7 and 8 days after surgery respectively (one required additional surgery), two abscesses (one treated by surgery), four cases of acute urinary retention requiring catheterization, two cases of faecal impaction and one patient reoperated on for anal fissure. Ten patients had postoperative external haemorrhoidal thrombosis, which required level-3 analgesics in three patients. We note that among the seven patients with anticoagulants or clopidogrel, no bleeding was reported. All in all, three patients were reoperated on for complications. Ninety-seven patients (75%) had no complications, without any level-3 analgesics or repeat surgery for recurrence. No significant anal incontinence was reported before or after surgery.

### Risks factors of a new haemorrhoidal surgery and having high postoperative pain

We analysed the risk factors for having new haemorrhoidal surgery for recurrence, a hard endpoint of failure. In univariate analysis (Table 3), female gender (OR 5.237 [1.199–30.26]) and presence of pruritus ani at baseline (OR 5.116 [1.167–24.33]) were associated with a risk of reoperation. In multivariate analyses, only pruritus ani at baseline was associated with reoperation (OR 5.8 [1.16–36.4]) (Table 4).

**Table 3:** Univariate analysis of risks factor of new haemorrhoidal surgery (Milligan–Morgan haemorrhoidectomy). Included patients with data (*N* = 126)

Variables		Univariate analysis	
Factor	Comparison	Odds ratio [95% CI]	Global *p* value
Age at surgery	≥ 50 years vs. < 50 years	0.511 [0.089–2.214]	0.3957
Sex	Female vs. Male	5.237 [1.199–30.26]	0.0373
Indication for surgery	Prolapsus vs. Bleeding	3.640 [0.652–23.53]	0.1807
	Prolapsus and bleeding vs. Bleeding	0.820 [0.122–5.500]	0.1807
Haemorrhoid grade	Grade III/IV vs. Grade I/II	3.487 [0.702–34.19]	0.1804
HEMO-FISS-QoL score at baseline	> 17 vs. ≤ 17	1.286 [0.300–5.972]	0.7348
Global anal discomfort at baseline	> 5 vs. ≤ 5	2.867 [0.660–16.49]	0.1837
Prolapsus at baseline	Yes vs. No	0.423 [0.077–4.337]	0.3888
Bleeding at baseline	Yes vs. No	0.409 [0.090–2.412]	0.2749
Dyschezia at baseline	Yes vs. No	3.214 [0.538–14.85]	0.1604
Pruritus ani at baseline	Yes vs. No	5.116 [1.167–24.33]	0.0312
Anal pain at baseline	Yes vs. No	0.870 [0.151–3.793]	0.8607
Number of joules	1 J	1.000 [0.999–1.000]	0.5483

Firth’s penalised logistic regression models the probability of being treated with Milligan–Morgan

**Table 4:** Multivariate analysis of risks factor of new haemorrhoidal surgery (Milligan–Morgan haemorrhoidectomy). Included patients with data (*N* = 126)

Variables		Multivariate analysis		
Item	Comparison	Odds ratio	95% CI	Global *p* value
Sex	Female vs. Male	4.659	[0.975–30.285]	0.0555
Indication for surgery	Prolapsus vs. Bleeding	3.742	[0.520–34.128]	0.0966
Indication for surgery	Prolapsus and bleeding vs. Bleeding	0.417	[0.039–3.815]	0.0966
Haemorrhoid grade	Grade III/IV vs. Grade I/II	3.430	[0.460–44.741]	0.2391
Global anal discomfort at baseline	> 5 vs. ≤ 5	2.688	[0.513–17.416]	0.2397
Dyschezia	Yes vs. No	1.862	[0.234–11.416]	0.5124
Pruritus ani at baseline	Yes vs. No	5.872	[1.161–36.401]	0.0312

Adjusted hazard ratio on age, sex, indication for surgery, haemorrhoid grade, HEMO-FISS-QoL score, anal discomfort, prolapsus and bleeding

We defined high postoperative pain as corresponding to at least one episode of mean pain level of  ≥ 6/10 and/or use of level-3 analgesics (30 patients). In univariate analyses, increased risk of such pain was associated with a global HEMO-FISS-QoL score at baseline > 17/100 (OR 3.476 [1.476–8.844]), a HEMO-FISS-QoL physical disorder dimension > 17/100 (OR 4.482 [1.862–11.90]), a HEMO-FISS-QoL psychology dimension > 17/100 (OR 2.623 [1.138–6.095]), pruritus ani (OR 3.423 [1.383–8.487]) and anal pain (OR 2.455 [1.067–5.689]) at baseline (Table 5). In multivariate analyses, the only two independent associated factors were a global HEMO-FISS-QoL score at baseline > 17/100 and pruritus ani at baseline (OR 3 [1.08–9.12] and 3.6 [1.23–10.89] respectively) (Table 6). When we analysed the dimension of the HEMO-FISS-QoL in a new multivariate analysis, only a HEMO-FISS-QoL physical disorder dimension > 17 and pruritus ani were associated with an increased risk of high postoperative pain with a 3.33 odds ratio [1.20–10.54] and 3.46 [1.158–10.547] respectively.

**Table 5:** Univariate analysis of risks factor of having high postoperative pain (mean pain ≥ 6/10 and/or use of level-3 antalgics). Included patients with data (*N* = 126)

Variables		Univariate analysis	
Factor	Comparison	Odds ratio [95% CI]	Global *p* value
Age at surgery	≥ 50 years vs. < 50 years	0.615 [0.262–1.393]	0.2543
Sex	Female vs. Male	1.349 [0.567–3.124]	0.4926
Indication for surgery	Prolapsus vs. Bleeding	0.147 [0.015–0.666]	0.0843
	Prolapsus and bleeding vs. Bleeding	0.579 [0.244–1.354]	0.0843
Haemorrhoid grade	Grade III/IV vs. Grade I/II	0.969 [0.430–2.220]	0.9408
HEMO-FISS-QoL score at baseline	> 17 vs. ≤ 17	3.476 [1.476–8.844]	0.0063
HEMO-FISS-QoL physical disorders dimension	> 17 vs. ≤ 17	4.482 [1.862–11.90]	0.0015
HEMO-FISS-QoL psychology dimension	> 17 vs. ≤ 17	2.623 [1.138–6.095]	0.0248
HEMO-FISS-QoL defecation dimension	> 17 vs. ≤ 17	1.861 [0.709–5.667]	0.2399
HEMO-FISS-QoL sexuality dimension	> 17 vs. ≤ 17	1.498 [0.656–3.404]	0.3374
Global anal discomfort at baseline	> 5 vs. ≤ 5	2.068 [0.884–4.979]	0.0996
Prolapsus at baseline	Yes vs. No	0.341 [0.100–1.198]	0.0954
Bleeding at baseline	Yes vs. No	2.757 [0.800–14.42]	0.1618
Dyschezia at baseline	Yes vs. No	1.548 [0.476–4.547]	0.4521
Pruritus ani at baseline	Yes vs. No	3.423 [1.383–8.487]	0.0083
Anal pain at baseline	Yes vs. No	2.455 [1.067–5.689]	0.0361
Number of joules	1 J	1.000 [1.000–1.000]	0.8910

Firth’s penalised logistic regression models the probability of being treated with Milligan–Morgan

**Table 6:** Multivariate analysis of risks factor of having high postoperative pain (mean pain  ≥ 6/10 and/or use of level 3 antalgics). Included patients with data (*N* = 126)

Variables		Multivariate analysis			
Item	Comparison	Odds ratio	95% CI	*p* value	Global *p* value
Indication for surgery	Prolapsus vs. Bleeding	0.097	[0.006–0.822]	0.0899	0.1080
Indication for surgery	Prolapsus and bleeding vs. Bleeding	0.449	[0.146–1.314]	0.5896	0.1080
HEMO-FISS-QoL score at baseline	> 17 vs. ≤ 17	3.009	[1.082–9.120]	0.0422	0.0422
Global anal discomfort at baseline	> 5 vs. ≤ 5	1.747	[0.636–4.921]	0.2855	0.2855
Prolapsus at baseline	Yes vs. No	0.626	[0.104–4.289]	0.6271	0.6271
Bleeding at baseline	Yes vs. No	0.436	[0.066–3.545]	0.4060	0.4060
Pruritus ani at baseline	Yes vs. No	3.622	[1.235–10.894]	0.0225	0.0225
Anal pain at baseline	Yes vs. No	1.643	[0.567–4.696]	0.3617	0.3617

Adjusted hazard ratio on age, sex, indication for surgery, haemorrhoid grade, HEMO-FISS-QoL score, anal discomfort, prolapsus and bleeding

## Discussion

We reported a prospective multicentre evaluation of RFA for the treatment of haemorrhoids in 129 patients with 1 year’s follow-up. RFA is associated with a significant improvement in the HEMO-FISS-QoL score, prolapse, bleeding and global anal discomfort at 3 months, and continued to show a high degree of patient satisfaction at 1 year. As expected from a minimally invasive technique, we observed mild postoperative pain for most patients and short medical leave (median 4 days). However, intense pain requiring level-3 analgesics was observed in 11 patients (8.5%) and patients should be warned of this possibility before surgery. Regarding safety, we observed the usual proctological surgery complications with three patients (2.3%) having to be reoperated on for complications. A specific complication seemed to be painful postoperative external thrombosis (10 patients, or 7.7%). Unexpectedly, having pruritus ani at baseline was the only risk factor found to be associated with reoperation by haemorrhoidectomy in multivariate analysis. This analysis must be interpreted with caution because of the lack of power reflected by the wide confidence intervals observed. One explanation of this observation could be an association of pruritus ani with external haemorrhoidal disease, which is not treated by radiofrequency and therefore a source of reoperation by classic haemorrhoidectomy. Pruritus ani and a baseline HEMO-FISS-QoL score > 17/100 were found to be factors associated with severe postoperative pain in multivariate analysis. Regarding pain, it is known that pruritus leads to central sensitisation and allokinesis or hyperkinesis [18]. Evaluation of the HEMO-FISS-QoL score at baseline, specifically the physical disorders score, may be useful in determining the patients most at risk for postoperative pain.

Other studies on RFA are mostly retrospective and monocentric [9–12]. Only Schäfer’s team has conducted two prospective studies [13, 14], the most recent of which is bicentric with 98 patients and 2 years’ follow-up [14]. As in our study, RFA was associated with significant symptom reduction and excellent patient satisfaction, low postoperative pain for most patients and on average few days of medical leave. The need for further treatment of haemorrhoids (instrumental or surgical) is a robust indicator of failure. In our study, 7 (5.4%) were reoperated on using Milligan–Morgan haemorrhoidectomy and 3 (2.3%) had infrared treatment. Tolksdorf et al. [14] reported the need for further surgery in 13% of patients (one underwent a stapled haemorrhoidopexy and 12 patients required rubber band ligatures after 2 year follow-up. Tolksdorf et al. [14] treated most of their patients under local anaesthesia while our patients were operated on under general or locoregional anaesthesia. It should be noted that these authors only included patients with a maximum of one or two pathological bundles, whereas in our studies, a median of 3 bundles were treated because the goal was to treat patients with more severe haemorrhoids in one session. In addition, they observed two bleeding episodes per procedure requiring suturing during the intervention [14].  The complication profile found in other studies is similar to our findings.

Our results can be compared with a meta-analysis comparing trans-anal haemorrhoidal dearterialisation with mucopexy (THDm) versus open haemorrhoidectomy [19]. The rate of reoperation for recurrence in our study was close to that of THDm (5.4% and 4.5% respectively) and higher than the rate observed with open haemorrhoidectomy (2.4%). The average medical leave time in our study was lower than with THDm (8 vs. 11 days on average) and considerably lower than that of open haemorrhoidectomy (22 days). Regarding average operating times, RFA is more rapid (15 min) than other techniques (30 min and 23 min for THDm and open haemorrhoidectomy respectively).

The strengths of our study are its prospective and multicentre character, quality of data collection and use of a validated quality of life score as primary assessment criterion. Weaknesses are the lack of comparison with other techniques, a relatively short follow-up interval, as well as few patients on anticoagulants and absence of RFA under local anaesthesia.

For clinicians and policy makers, this study indicates that RFA is safe and effective and should be recognised by the health authorities in order to validate reimbursement. For researchers, this study indicates that there is still potential for surgical innovation for this old pathology.

In conclusion, this is the largest prospective multicentre study on RFA for haemorrhoidal disease involving 129 patients with 1 year follow-up. In patients with grade II–III haemorrhoids without external disease, and after failure of medico-instrumental therapy, this approach provides an excellent efficacy and safety profile that meets the expectations of a minimally invasive technique. Longer-term and randomised studies are needed to confirm our findings. Two clinical trials are currently under way and will compare RFA versus haemorrhoid artery ligation (HAL) Doppler and versus laser.

## Supplementary Information

Below is the link to the electronic supplementary material.

Supplementary file1 (DOCX 17 KB)

## Data Availability

The data that support the findings of this study are available from the corresponding author, [AL], upon reasonable request.
