# Supplementary material for: French multicentre prospective evaluation of radiofrequency ablation in the management of haemorrhoidal disease
Source: Tech Coloproctol. 2023 Apr 2;27(10):873–83. doi: 10.1007/s10151-023-02787-1 (PMC10485119; doi:10.1007/s10151-023-02787-1)
Supplement: Supplementary file 1 — Supplementary file1 (DOCX 17 KB) [file 10151_2023_2787_MOESM1_ESM.docx]

Supplementary table: Patients recruited per centre and effect of centre on the HEMO-FISS-QoL score at 3 months.

1. Patients recruited per centre.

| **Centre** | N | % |
| --- | --- | --- |
| Total | 129 |  |
| 01 | 5 | (3.9) |
| 02 | 2 | (1.6) |
| 03 | 4 | (3.1) |
| 05 | 4 | (3.1) |
| 06 | 9 | (7.0) |
| 07 | 9 | (7.0) |
| 09 | 4 | (3.1) |
| 10 | 11 | (8.5) |
| 11 | 4 | (3.1) |
| 12 | 10 | (7.8) |
| 14 | 40 | (31.0) |
| 15 | 3 | (2.3) |
| 16 | 4 | (3.1) |
| 18 | 6 | (4.7) |
| 19 | 11 | (8.5) |
| 21 | 3 | (2.3) |

1. Description of the effect of the centre on the HEMO-FISS-QoL score 3 months after the surgery in a general linear model (ANCOVA)- All included patients treated (N=129). Effect of centre 14 and the HEMO-FISS-QoL score before surgery was tested on the HEMO-FISS-QoL score 3 months after surgery.

|  | **Reference** | **estimate [IC 95%]** | | **P-value** |
| --- | --- | --- | --- | --- |
| Other centres | Centre 14 | 2.861 | [-1.339,7.062] | 0.181 |
